# Effectiveness of lyophilized growth factors injection for subacromial impingement syndrome: a prospective randomized double-blind placebo-controlled study

**DOI:** 10.1186/s13018-023-03548-4

**Published:** 2023-01-31

**Authors:** Sherine Mahmoud El-Sherif, Mowaffak Moustafa Abdel-Hamid, Jailan Mohamed Ashraf Mohamed Noureldin, Hossam Moustafa Fahmy, Hoda Mohamed Aly Abdel-Naby

**Affiliations:** 1 Present Address: Department Physical Medicine, Rheumatology and Rehabilitation, Faculty of Medicine, Alexandria University, Medaan El-Khartoom Square, Al-Azaritah, Alexandria, Egypt; 2 Clinical Pathology, Faculty of Medicine, Ain Shams University, Cairo, Egypt

**Keywords:** Shoulder impingement, Lyophilized growth factors, Subacromial injection, SPADI, Painful arc

## Abstract

**Background:**

Platelet-derived lyophilized growth factors (L-GFs) use a standardized number of allogenic pathogen-free platelets instead of autologous platelets used in PRP as a source of growth factors. This study aimed to evaluate the efficacy of L-GF injection versus placebo in subacromial impingement (SIS) treatment.

**Methods:**

The current randomized double-blind placebo-controlled study included sixty patients (40 females and 20 males, aged between 24 and 75 years) diagnosed with SIS (both clinically and sonographically). Patients were randomly assigned to two equal groups. Under ultrasound guidance, group 1 received subacromial saline injection, and group 2 received L-GF injection. Clinical examination, pain visual analogue scale (VAS), shoulder pain and disability index (SPADI) and shoulder ultrasound were performed before and at the 8th week after injection.

**Results:**

Follow-up assessment showed statistically significant improvement in the L-GF group regarding active flexion, active and passive internal rotation and extension, SPADI-disability scale, VAS and thickness of the supraspinatus tendon by US. Regression analysis showed that group 1 was approximately 30 times more likely than the L-GF group to experience painful arc at follow-up. Both groups showed statistically significant improvement in SPADI-pain scale and SPADI-total, flexion and abduction (still the mean value of abduction was significantly higher in the L-GF group).

**Conclusions:**

L-GF injection resulted in clinically significant reductions in pain and functional disability outcomes in patients with SIS. An objective significant reduction in the thickness of the supraspinatus tendon, measured by ultrasound, in the L-GF group hopefully encourages proper healing and functioning in SIS.

*Trial registration***:** The identification number is NCT04330027, date of first registration (01/04/2020). Unique on 21/11/2019, Protocol ID: 0106178.

## Key points

1. L-GF uses a standardized number of allogenic pathogen-free platelets instead of autologous platelets used in PRP as a source of growth factors.
2. L-GF injection in patients with SIS has resulted in significant improvement both clinically and sonographically, signifying its potential beneficial effects on tendon healing.
3. L-GF injection could be a mini-invasive intervention for SIS treatment that gives hopeful results.

## Background

Shoulder pain is the third leading musculoskeletal complaint with subacromial impingement syndrome (SIS), accounting for 44–65% of all shoulder complaints, resulting in functional limitation and disability [1–4].

SIS is mechanical compression of the rotator cuff tendons, long head of the biceps and subacromial bursae under the coracoacromial arch. [5] It is an umbrella term including rotator cuff tendonitis, tendinosis, partial or complete tears and bursal inflammation [3–7]. It could be due to acromioclavicular joint arthritis, acromial spurs, rotator cuff tendinopathy and scapular muscular dyskinesia. This results in a reduction in the potential space available for rotator cuff tendons in the subacromial space and triggers the cascade associated with SIS [1, 5].

SIS is diagnosed clinically as localized anterolateral shoulder pain lasting longer than 2 weeks, exacerbated by arm elevation, above-head movement and lying on the affected side [6, 8]. The “impingement sign” manifests as pain on shoulder abduction between 70° and 120° (painful arc) and is confirmed with the “impingement test”, where pain is relieved by injection of 5–10 ml of 1% xylocaine into the subacromial space. Provocative tests such as Neer, Hawkins and empty can tests can be positive [8].

There is no consensus regarding effective treatment for SIS. This lack of consensus might be related to a proposed multifactorial pathogenesis [3, 5]. Nevertheless, conservative therapy, including exercise, analgesia, physiotherapy and subacromial injections, is the first-line treatment and yields satisfactory results [5, 9]. Alternatively, those who have not improved resort to surgery [5]. Subacromial corticosteroid injection is commonly used; however, its efficacy remains controversial [10–12]. On the other hand, platelet-rich plasma (PRP) injection has recently garnered significant attention in stimulating tissue healing in numerous musculotendinous pathologies, including SIS [13–16].

The regenerative and immunomodulatory properties of PRP depend on the hyperphysiological content of autologous growth factors released after platelet activation, including platelet-derived, transforming, fibroblast, endothelial, connective tissue, vascular endothelial and hepatocyte growth factors (HGFs), which stimulate epithelial cells and fibroblasts, resulting in new collagen and elastin formation [15–17].

Significant variability exists among different PRP preparations, whether commercially available or non-commercial double-centrifugation techniques, with variation in the number of platelets and concentration of growth factors. The lack of standardization for an ideal number of platelets has a definite influence on clinical results [15–17].

A new patented blood product named lyophilized growth factors (L-GF) [18], which is a refined modification of conventional PRP therapy, has been developed. It uses allogenic pathogen-free platelets with a standardized number instead of autologous platelets used in PRP as a source of growth factors [19]. It showed encouraging results in patients with primary knee osteoarthritis with regard to pain, effusion and function [19], in addition to efficacy and safety in plantar fasciitis [20]. This growing evidence supporting its safety and efficacy encourages further research to verify its utility as a treatment for a spectrum of musculoskeletal disorders, including SIS.

The aim of this study was to evaluate the efficacy of injection of platelet-derived L-GF in the treatment of subacromial impingement.

### Subjects & methods

This double-blind, randomized placebo-controlled trial was conducted on all consecutive patients with SIS who did not respond to conservative treatment for more than 3 months and were recruited from the Outpatient Clinic of Physical Medicine, Rheumatology and Rehabilitation Department, Alexandria University Hospitals between October 2020 and March 2021. The trial registration number is NCT04330027, date of first registration (01/04/2020).

*Diagnosis of SIS* Patients were diagnosed with SIS by the following:

*Clinically* Anterolateral shoulder and/or lateral upper arm pain, [6] positive painful arc between 70° and 120° of active abduction, [8] positive impingement sign (Neer’s sign or Hawkins–Kennedy test). [3]

*Ultrasonographically* Elicitation of the transient arc of pain during shoulder abduction, coinciding with passage of the supraspinatus tendon beneath the coracoacromial arch. [21]

*Exclusion criteria included patients with a* history of shoulder surgery, fracture, dislocation or subluxation, full-thickness rotator cuff tear, positive “drop arm sign”, frozen shoulder or degenerative arthropathy of the glenohumeral joint, cervical spine or upper extremity disorders with a significant impact on the shoulder, diabetes mellitus, active infection or other painful, function-limiting disorders of the shoulder and significant systemic disease [2, 4, 14, 19, 20].

Approval for this prospective clinical study was granted by the local ethics committee, and informed consent was obtained from all patients.

The current study included 60 patients (40 females and 20 males, aged between 24 and 75 years) who were randomly divided into two equal groups. Group 1 received subacromial saline injection, and group 2 received L-GF injection. Both groups were injected once under US guidance.

## Baseline assessment

### Clinical examination

Assess for tender points, provocative tests (painful arc, Neer’s sign, Hawkins–Kennedy and empty can tests), shoulder ROM (active and passive) using a goniometer, muscle power around the shoulder using Medical Research Council grading, Visual analogue scale of pain (VAS) and shoulder pain and disability index (SPADI).

### Ultrasonographic assessment

Using a 3–16 MHz linear array transducer (HS50; Samsung Medicine, Seoul, Korea), the supraspinatus tendon thickness of the affected shoulder was measured initially in both longitudinal (at the medial edge of the footprint) and transverse views (two measurement sites, 5 and 10 mm posterior to the edge of the biceps tendon), and the average was used. [21, 22] Subacromial bursa as well as acromiohumeral distance in neutral and 90° shoulder abduction were measured as well as assessment for tendon tears.

### Patients randomization and blinded injection preparation

Patients were allocated by simple randomization by a trained nurse into two equal groups (30 patients each). The patients, clinical examiner and sonographer were blinded to the type of injection given. The nurse prepared syringes containing either saline (2 ml of 0.9% NaCl) or L-GF (dissolved in 1 ml saline and 1 ml lignocaine), wrapped them up using opaque adhesive tape to conceal their content and labeled them with a name code of the patients.

### L-GF preparation

L-GF was supplied as a powder in a tightly sealed container. The amount of growth factors in each vial was standardized, equivalent to those obtained from a platelet count of 106/μl found in 20 ml of whole blood.

The platelet concentrate was subjected to a first pathogen reduction step via UV irradiation using the Mirasol TM system that disrupts RNA and/or DNA of microorganisms, including enveloped, non-enveloped virus, bacteria, fungi, etc. Then, the platelets are activated to release part of the alpha granules and growth factors present therein, thereby providing a fluid platelet releasate, which is subjected to a fibrinogen reduction step to obtain a fibrinogen-depleted fluid platelet releasate (for easy administration in intralesional injections). A second confirmatory pathogen concentration reduction step was taken to disrupt enveloped viruses. Finally, sterile filtering of platelet releasate was performed, and a filtrate liquid containing growth factors was produced and freeze-dried to form a powder [19].

### Ultrasound-guided injection procedure

All interventional procedures were performed with patients seated, arm hanged by side, under complete aseptic conditions and upon real-time sonographic guidance. A 5-cm 21-gauge needle was inserted parallel to the long axis of the ultrasound transducer (lateral approach) and advanced under continuous guidance into the subacromial space.

Patients were instructed to avoid lifting weights on the affected limb for 2 weeks [14] and refrain from any NSAIDs for 1 month.

### Follow-up assessment at the 8th week

Clinical examination, VAS of pain, SPADI and US reassessment of the shoulder were performed. Blindness of the examiner and the patient to the injected preparation was maintained.

### Unblinding

**Fig. 1:**
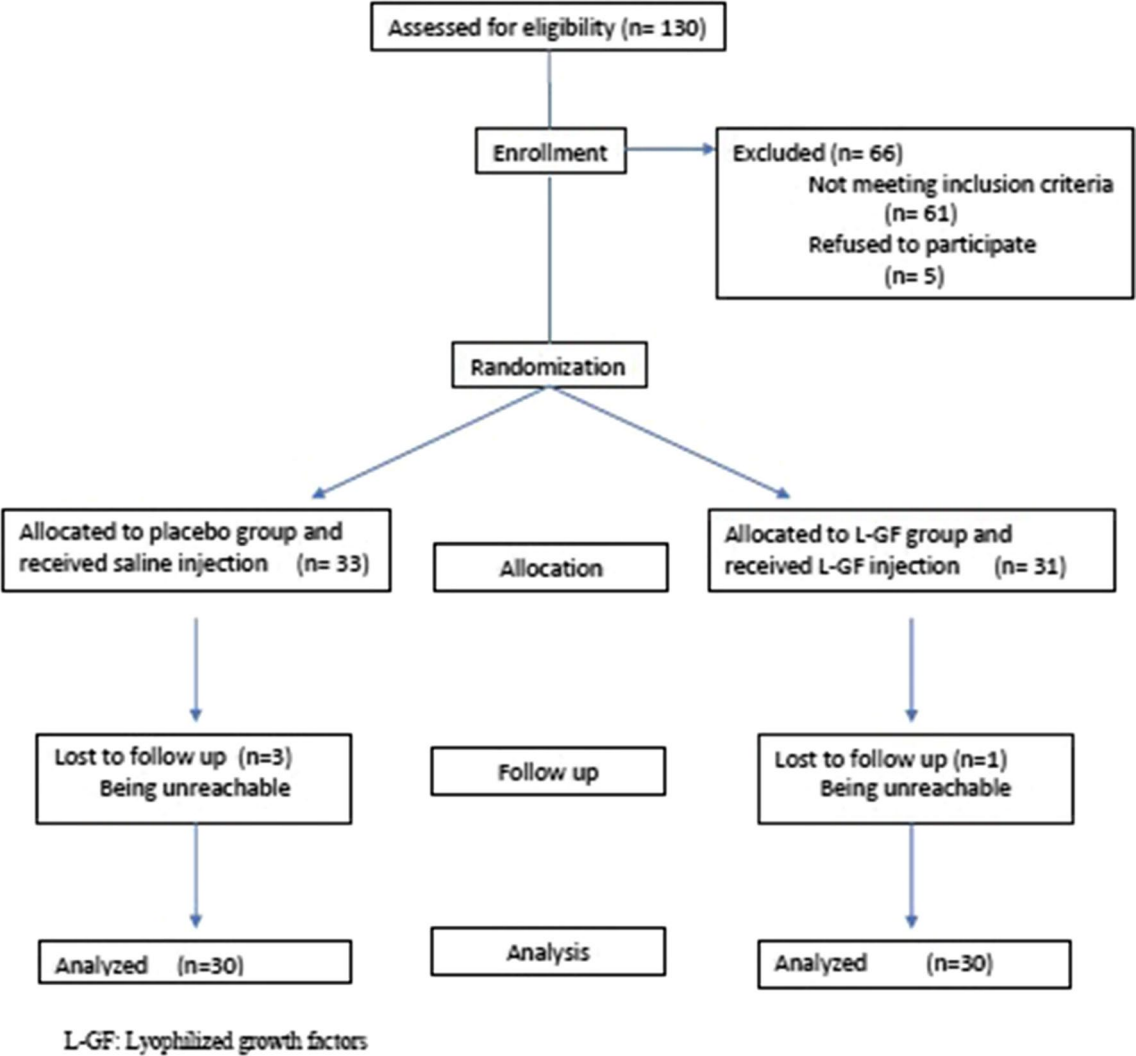
Patients’ disposition flow diagram (CONSORT flowchart)

At the end of the study, the trained nurse broke the code in the presence of the assessors. A CONSORT flowchart is also available, which displays the progress of all participants throughout the study (Fig. 1).

*Statistical analysis* Statistical analysis was conducted using IBM-SPSS software package version 20.0. Means and standard deviations were used to describe quantitative data. For normally distributed data, comparisons were performed by independent t tests. The significance was set at 5%. Logistic regression analysis of the relationship between a set of dependent and other independent variables was performed to identify prognostic factors.

Sample size was calculated using G-Power 3 software based on expected differences in pain and function outcomes in the studied groups. To achieve a power of 80% at the 95% confidence level, the minimum accepted sample size was 23 patients per group with a total of 46 patients.

## Results

Group 1 (the saline group, *n* = 30) included 17 females and 13 males with a mean age of 50 years (ranging from 27 to 75) and a mean disease duration of 16 ± 22 months. Group 2 (the L-GF group, *n* = 30) included 23 females and seven males with a mean age of 45 years (ranging from 24 to 70) and a mean disease duration of 18 ± 25 months. There was no statistically significant difference between the two groups regarding patients’ age, gender, disease duration, occupation, handedness, side affected and its dominance.

Regarding shoulder ROM assessment (Table 1), there was no statistically significant difference between both groups comparing baseline mean values of active and passive ROM in all directions. At follow-up, there was a statistically significant improvement in passive flexion and abduction in both groups, with a significantly higher mean value of abduction in the L-GF group. Active flexion as well as active and passive internal rotation and extension significantly improved only in the L-GF group.

**Table 1:** Comparing baseline and follow-up motion ranges within and between the two groups

ROM median (in degrees)		Saline group (*n* = 30)	L-GF group (*n* = 30)	p2
Active flexion	Baseline	152.5	160	0.141
	Follow-up	162.5	170	0.177
	p1	0.068	0.006*	
	% Change	2.5	2.5	0.684
Passive flexion	Baseline	160	167.5	0.084
	Follow-up	170	170	0.161
	p1	0.003*	0.021*	
	% Change	5	5	0.483
Active abduction	Baseline	150	160	0.228
	Follow-up	150	165	0.295
	p1	0.280	.125	
	% Change	0	5	0.823
Passive abduction	Baseline	160	162.5	0.191
	Follow-up	170	180	0.043*
	p1	< 0.001*	0.014*	
	Change	10	10	0.725
Active internal rotation	Baseline	75	80	0.613
	Follow-up	80	85	0.032*
	p1	0.172	< 0.001*	
	% Change	0	12.5	0.023*
Passive internal rotation	Baseline	77.5	85	0.321
	Follow-up	85	90	0.003*
	p1	0.255	0.001*	
	% Change	0	7.5	0.069
Active extension	Baseline	57.5	57.5	0.857
	Follow-up	60.00	60.00	0.178
	p1	0.055	0.002*	
	% Change	2.50	5.00	0.148
Passive extension	Baseline	60.00	60.00	0.915
	Follow-up	60.00	60.00	0.151
	p1	0.164	0.002*	
	% Change	0.00	5.00	0.084

p1: of paired samples *t* test, p2: of independent samples *t* test

*Statistically significant at *p* ≤ 0.05

At follow-up, both groups showed statistically significant improvement regarding VAS, SPADI-pain scale and SPADI-total mean values in both groups, but these variables were still significantly lower in the L-GF group at follow-up than in the saline group. Only the L-GF group showed statistically significant improvement regarding the SPADI-disability scale (Table 2).

**Table 2:** Baseline and follow-up VAS, SPADI-pain, -disability and -total scales within and between the two groups

		Saline group *n* = 30 mean ± SD	L-GF group *n* = 30 mean ± SD	t2	p2
VAS	Baseline	7.67 ± 1.92	6.90 ± 1.83	1.586	0.118
	Follow-up	5.87 ± 2.60	3.97 ± 2.57	2.851	0.006*
	t1	3.949	7.478		
	p1	< 0.001*	< 0.001*		
	% Change	1.80 ± 2.50	2.93 ± 2.15	− 1.884	0.065
SPADI-pain (%)	Baseline	69.37 ± 17.19	60.03 ± 16.75	2.130	0.037*
	Follow-up	55.95 ± 27.02	39.20 ± 24.26	2.527	0.014*
	t1	2.843	4.851		
	p1	0.008*	< 0.001*		
	% Change	13.42 ± 25.85	20.83 ± 23.52	− 1.162	0.250
SPADI-Disability (%)	Baseline	56.40 ± 19.35	47.73 ± 19.96	1.708	0.093
	Follow-up	48.50 ± 25.58	28.67 ± 20.52	3.312	0.002*
	t1	1.819	4.565		
	p1	0.079	< 0.001*		
	% Change	7.90 ± 23.77	19.05 ± 22.86	− 1.853	0.069
SPADI-total (%)	Baseline	61.28 ± 16.76	52.63 ± 16.65	2.006	0.050*
	Follow-up	51.36 ± 24.92	32.87 ± 20.61	3.132	0.003*
	t1	2.356	5.084		
	p1	0.025*	< 0.001*		
	% Change	9.27 ± 23.76	19.88 ± 21.09	-1.829	0.073

*VAS* visual analogue scale, *SPADI* shoulder pain and disability index, *SD* Standard deviation, t1: paired samples *t* test, t2: independent samples *t* test, p1: of paired samples *t* test, p2: of independent samples *t* test

*Statistically significant at *p* ≤ 0.05

Subacromial tenderness improved significantly at follow-up only in the L-GF group. However, bicipital and acromioclavicular joint tenderness as well as Neer’s and Hawkins–Kennedy sign and empty can test showed no statistically significant difference between baseline and follow-up in both groups.

As shown in Table 3, at follow-up, the frequency of cases with a negative painful arc increased significantly to 12 cases in the L-GF group compared to only one case in the saline group. In addition, the frequency of cases with improvement of SPADI-total score was significantly higher in the L-GF group (27 cases) compared to only 19 cases in the saline group. Table 3 presents the frequency of negative painful arc at follow-up (12 cases in L-GF & 1 case in saline group) as well as improvement in SPADI-total score (27 cases in L-GF & 19 cases in saline group), with a statistically significant difference between both groups.

**Table 3:** Painful arc and SPADI-total score percentage of improvement in the two groups at follow-up

	Comparison point	Saline group *n* = 30	L-GF group *n* = 30	*p*
Painful arc	Negative	1 (3%)	12 (40%)	0.001*
	Positive	29 (97%)	18 (60%)	
SPADI-total	Improvement	19 (63%)	27 (90%)	0.03*
	Worsening	11 (37%)	3 (10%)	

SPADI: Shoulder pain and disability index, p: Fischer’s exact test

*Statistically significant at p ≤ 0.05

Sonographic assessment, Table 4, displays statistically significant improvement of supraspinatus tendon thickness in longitudinal view at follow-up in the L-GF group. Otherwise, there was no statistically significant improvement in any of the other sonographic variables.

**Table 4:** Supraspinatus tendon thickness in the two groups at baseline and follow-up

		Saline group (*n* = 30) mean ± SD	L-GF group (*n* = 30) mean ± SD	*t*	*p*
US-L (mm)	Baseline Follow-up t1 p2	6.3 ± 1.1 6.2 ± 1.2 1.340 0.191	6.0 ± 1.3 5.8 ± 1.2 2.147 0.040*	0.933 1.132	0.355 0.262
US-T (mm)	Baseline Follow-up t1 p2	6.0 ± 1.3 5.7 ± 1.2 1.657 0.108	5.5 ± 1.1 5.2 ± 1.1 1.835 0.74	1.650 1.539	0.104 0.129

*US-L* ultrasonographic measurement of supraspinatus tendon thickness in longitudinal view, *US-T* ultrasonographic measurement of average supraspinatus tendon thickness in transverse view, t1: Paired samples *t* test, t2: Independent samples t test, p1: of paired samples *t* test, p2: of independent samples *t* test

*Statistically significant by *t* test, at *p* ≤ 0.05

*Adverse events* No major adverse events were reported in either group.

A multivariate stepwise logistic regression analysis showed that the saline group patients were 30 times more likely to experience persistent painful arc at follow-up than patients in the L-GF group (OR = 30.1, *p* = 0.008). Patients with baseline-positive Neer’s sign were 11 times more likely to have persistent painful arc at follow-up (OR = 10.8, *p* = 0.011) (Table 5). Additionally, with each 1-degree increase in passive extension ROM at baseline, there was a 0.85 probability that the patient would experience a painful arc at follow-up (OR = 0.85, *p* = 0.015). Moreover, with each increase in age by 1 year, there is a 1.09 probability that the patient will get a positive Hawkins–Kennedy test at follow-up (OR = 1.091, *p* = 0.016). In addition, patients with a thicker supraspinatus tendon in the transverse view at baseline were more likely to have a positive empty can test at follow-up.

**Table 5:** Logistic regression analysis between some independent variables and dependent variables at follow-up

	*p*	OR	Overall model p	Nagelkerke R square
*Predictors of persistent painful arc at follow-up*				
Saline injection	0.008*	30.110	< 0.001*	0.578
Baseline Neer’s sign	0.011*	10.8		
Baseline passive extension ROM	0.015*	0.852		
*Predictors of Neer’s sign at follow-up*				
Baseline Neer’s sign	< 0.001*	0.93	< 0.001*	0.295
*Predictors of empty can test at follow-up*				
Baseline US-T	0.025*	1785.07	0.007*	0.150
*Predictors of Hawkins–Kennedy sign at follow-up*				
Baseline Hawkins–Kennedy sign	0.039*	0.122	0.007*	0.237
Age	0.016*	1.091		

(Only the significant predictors are shown in table)

*US-T* ultrasonographic measurement of average supraspinatus tendon thickness in transverse view, *OR* odds ratio

*Statistically significant at *p* ≤ 0.05

Table 6 presents multiple linear stepwise regression models predicting VAS, SPADI-total and thickness of the supraspinatus tendon (longitudinal and transverse views) at follow-up. Significantly lower VAS at follow-up in the L-GF group was found (*b* = − 1.38, *p* = 0.025), with higher values in patients with higher baseline VAS (*b* = 0.67, *p* < 0.001). Similarly, SPADI-total was significantly lower in the L-GF group (*b* = − 13.73, p = 0.16), with higher values in right-handed patients and those with higher baseline SPADI-total (*p* < 0.001). In addition, patients with a positive Hawkins–Kennedy test at baseline were more likely than those with a negative test to have a thicker supraspinatus tendon in the longitudinal view at follow-up (b = 0.62, p = 0.016). Furthermore, patients with a thicker supraspinatus tendon, in both longitudinal and transverse views, at baseline were more likely to have a thicker tendon at follow-up (*b* = 0.932, *p* < 0.001 and *b* = 0.715, *p* < 0.001, respectively).

**Table 6:** Linear regression analysis of predictors of VAS, SPADI-total and supraspinatus tendon thickness at follow-up

	*p*	OR	Overall model F	Overall model p	R square
*Predictors of follow-up VAS*					
Baseline VAS	< 0.001*	0.67	14.3	< 0.001*	0.334
L-GF injection	0.025*	− 1.38			
*Predictors of follow-up SPADI-total*					
Baseline SPADI-total	< 0.001*	0.616	9.298	< 0.001*	0.332
L-GF injection	0.016*	− 13.73			
Handedness	0.045*	31.40			
*Predictors of follow-up US-L*					
Baseline US-L	< 0.001*	0.932	117.52	< 0.001*	0.805
Baseline Hawkins- Kennedy test	0.016*	0.062			
*Predictors of follow-up US-T*					
Baseline US-T	< 0.001*	0.715	36.677	< 0.001*	0.563

(Only the significant predictors are shown in table)

*VAS* visual analogue scale, *SPADI* shoulder pain and disability index, *US-L, -T* ultrasonographic measurement of supraspinatus tendon thickness in longitudinal and transverse views, respectively, *OR* odds ratio

*Statistically significant at *p* ≤ 0.05

## Discussion

The regenerative effect of L-GF in SIS was tested on different outcome measures (clinical and sonographic). Pain has been considered one of the main outcome measures and has shown significantly more improvement in the L-GF group (2.9 points in mean VAS) than in the saline group (1.8 points), which represents a 42% reduction in the former versus 23% in the latter. Linear regression also showed that L-GF injection was a significant predictor of lower follow-up VAS scores, and controlling for other variables, follow-up VAS scores were significantly lower in the L-GF group than in the saline group by 1.38 points. In addition, subacromial tenderness was elicited in a significantly lower number of patients in the L-GF group at follow-up than in the saline group. This effect of L-GF on pain reduction could be explained by the anti-inflammatory effect of HGF released from platelets during the activation step [17, 24]. In a study on both rabbit tendon cells (in vitro) and mouse Achilles tendons (in vivo), PRP and HGF were found to suppress the expression of COX-1, COX-2 and PGE2, which are major markers of inflammation. These results were confirmed by the administration of HGF antibody with PRP or HGF, which blocked the suppression of inflammation [24]. Other studies have proven that this anti-inflammatory effect occurs through suppression of NF-kappa B activation [25].

Limited ROM is caused by altered shoulder kinematics [26], among which biomechanical factors such as posterior capsule tightness lead to deficits in glenohumeral internal rotation, thus predisposing patients to SIS [27]. There was significant improvement among the L-GF group in active and passive internal rotation within 8 weeks, which was lacking in the saline group. This is probably caused by growth factors, which may have the potential to counteract the consequences of a tight posterior capsule [28]. There was also significant restoration of active and passive shoulder flexion and extension in the L-GF group but not in the saline group. Thus, suppression of fibrous formations might have induced a positive effect on improving tendon glide and, hence, ROM. Active movement imposes tensile stress on muscle fibers and their gliding tendon [28], so when inflamed or injured, pain is encountered. On the other hand, when no pain is encountered during active movement, this means that tissues have restored their physiological working capacity. A number of studies have demonstrated this effect of PRP in improving shoulder ROM in adhesive capsulitis [24, 28].

For the painful arc sign, logistic regression showed that L-GF was a significant predictor of a negative painful arc sign at follow-up (40% in L-GF versus 3% in saline group). Furthermore, by adjusting for covariates, the L-GF group was 30 times more likely to have a negative painful arc at follow-up than the saline group. This suggests that L-GF, through its anti-inflammatory effect, may have acted by inducing considerable pain relief as well as decreasing supraspinatus tendon thickness, thus improving the impingement while passing under the coracoacromial arch during abduction. In comparison with steroid injection, a study comparing the effectiveness of steroids to local anesthetic injections for supraspinatus tears found considerable pain relief after steroid injection, yet no improvement regarding the painful arc sign [31].

To evaluate patients’ pain during different activities and the resulting disability, SPADI-total and subscores were assessed at baseline and follow-up. The SPADI-total and SPADI-pain subscales showed a statistically significant reduction in both groups. However, the magnitude of improvement was larger in the L-GF group than in the saline group. Additionally, linear regression revealed that L-GF was a significant predictor of a lower SPADI-total score at follow-up. For the SPADI-disability subscale, only the L-GF group showed statistically significant improvement.

In agreement with our results, a meta-analysis has shown significantly better SPADI scores at 3 weeks, 3 months and 6 months among patients treated with PRP than with saline, exercise therapy or dry needling [32]. On the other hand, a randomized placebo-controlled study showed no significant difference between PRP and saline with regard to SPADI in rotator cuff tendinopathy after 1 year. This could be due to a lack of standardization in PRP preparation [33].

To objectively evaluate soft tissue organization, ultrasonography was used at follow-up to measure the thickness of the supraspinatus tendon. A statistically significant decrease in tendon longitudinal thickness was detected in the L-GF group only. A decrease toward normal thickness is considered a sign of successful tendon regeneration where a more organized fibrillar pattern is restored since L-GF enhances tendon remodeling [34]. Lind et al. [35] reported a similar decrease in Achilles tendon thickness after injection of polidocanol. Another study found no significant change in tendon thickness after needle tenotomy and PRP injection in cases of chronic tendinopathies. This could be explained by the variability of the injected tendons and their characteristics [36].

Although 17 months was the mean duration of symptoms in our patients, which poses great challenges to healing, this chronicity still did not affect the outcome measures in the L-GF group, as concluded by the regression analysis. Another challenge was the age of our patients, which is a well-known risk factor for degenerative pathologies [29]. An age-dependent decrease in the vascular supply of the supraspinatus tendon was seen by contrast-enhanced ultrasonography, which impacts the tendon’s healing capacity [30]. This supports the wider use of growth and regenerative factors in tendinopathies aiming at rapid repair and functional improvement. [13, 24, 32, 37]

## Conclusions

L-GF injection in patients with SIS resulted in significant improvement in pain and disability according to the improvement in shoulder range of movement, painful arc sign, VAS, SPADI, as well as significant reduction in the thickness of the supraspinatus tendon as measured by ultrasound compared to the saline group.

### Strengths and limitations

The major strengths of this study are the presence of a control group and strict application of double-blinding. A significant limitation was the single vial of L-GF injection, which might have affected the outcome if more dosage was used.

## Data Availability

The datasets used and/or analyzed during the current study are available from the corresponding author on reasonable request.

## Abbreviations

HGF: Hepatocyte growth factor
L-GF: Lyophilized growth factors
NSAIDs: Nonsteroidal anti-inflammatory drugs
PRP: Platelet-rich plasma
SIS: Shoulder impingement syndrome
SPADI: Shoulder pain and disability index
US: Ultrasonography
VAS: Visual analogue scale
